# Patent Medicines

**Published:** 1905-02

**Authors:** 


### Patent Medicines.

A meeting of the Proprietary Association of Canada is advertised to take place in Toronto on the 7th of September. The Honorable George T. Fulford, Senator, of Brockville, is the Honorary President of this body of scientists and philanthropists. The commodities in which the members of this association deal are not to be confounded with the pharmaceutical preparations manufactured for the profession by those firms whose names are a guarantee of purity and excellence. Their products are best described by the terms nostrums or patent medicines. There are certain subjects which we would commend for the consideration of this association, and we should like to be informed as to the results of their deliberations. A paper might be read upon the inadvisability of employing alcohol to the amount of 50 per cent in the preparations. There might be a discussion upon the pharmacological effects of large doses of opium when given to children, upon the use of cocaine in catarrhal conditions, and upon the employment of abortifacients. An exhibition might be made of large volumes containing cuttings from the newspapers to illustrate the methods of advertising which some owners of patent medicines employ. There might also be a Medico-Legal Section in which the relation of the procedure of these advertisers to the criminal law would be a profitable subject for consideration. It is not unlikely that some day this last subject of criminal responsibility will receive attention at the hands of a body which will not deal with it in so liberal a spirit as the members of the Proprietary Association would be disposed to manifest. —*Montreal Med. Jour.*

